# New Fusion Transcripts Identified in Normal Karyotype Acute Myeloid Leukemia

**DOI:** 10.1371/journal.pone.0051203

**Published:** 2012-12-12

**Authors:** Hongxiu Wen, Yongjin Li, Sami N. Malek, Yeong C. Kim, Jia Xu, Peixian Chen, Fengxia Xiao, Xin Huang, Xianzheng Zhou, Zhenyu Xuan, Shiva Mankala, Guihua Hou, Janet D. Rowley, Michael Q. Zhang, San Ming Wang

**Affiliations:** 1 Department of Genetics, Cell Biology and Anatomy, University of Nebraska Medical Center, Omaha, Nebraska, United States of America; 2 Department of Molecular and Cell Biology, The University of Texas at Dallas, Richardson, Texas, United States of America; 3 Department of Medicine, University of Michigan Medical Center, Ann Arbor, Michigan, United States of America; 4 Institute of Experimental Nuclear Medicine, Shandong University School of Medicine, Jinan, China; 5 Department of Medicine, University of Nebraska Medical Center, Omaha, Nebraska, United States of America; 6 School of Medicine, New York Medical College, New York, United States of America; 7 Department of Medicine, University of Chicago Medical Center, Chicago, Illinois, United States of America; 8 Eppley Cancer Institute, University of Nebraska Medical Center, Omaha, Nebraska, United States of America; West Virginia University School of Medicine, United States of America

## Abstract

Genetic aberrations contribute to acute myeloid leukemia (AML). However, half of AML cases do not contain the well-known aberrations detectable mostly by cytogenetic analysis, and these cases are classified as normal karyotype AML. Different outcomes of normal karyotype AML suggest that this subgroup of AML could be genetically heterogeneous. But lack of genetic markers makes it difficult to further study this subgroup of AML. Using paired-end RNAseq method, we performed a transcriptome analysis in 45 AML cases including 29 normal karyotype AML, 8 abnormal karyotype AML and 8 AML without karyotype informaiton. Our study identified 134 fusion transcripts, all of which were formed between the partner genes adjacent in the same chromosome and distributed at different frequencies in the AML cases. Seven fusions are exclusively present in normal karyotype AML, and the rest fusions are shared between the normal karyotype AML and abnormal karyotype AML. *CIITA*, a master regulator of MHC class II gene expression and truncated in B-cell lymphoma and Hodgkin disease, is found to fuse with *DEXI* in 48% of normal karyotype AML cases. The fusion transcripts formed between adjacent genes highlight the possibility that certain such fusions could be involved in oncological process in AML, and provide a new source to identify genetic markers for normal karyotype AML.

## Introduction

Acute myeloid leukemia (AML) is a major type of leukemia, with estimated 13,780 new cases and 10,200 death in the United States in 2012 (American Cancer Society, 2012, http://www.cancer.org/Research/CancerFactsFigures/CancerFactsFigures/cancer-facts-figures-2012). Genetic aberrations including translocation, amplification, inversion, insertion, and deletion, are well-known to contribute to leukemia [1], and multiple recurrent genetic aberrations including t(9;11), inv(16), t(15;17) and t(8;21), have been identified in AML. These aberrations disrupt the normal structure of the affected genes and form fusion genes. The roles of these fusion genes in promoting myeloid leukemogenesis have been extensively studied. The disrupted genetic structures are widely used clinically as specific markers for diagnosis, prognosis, and treatment of AML [2].

Nearly half of AML cases do not contain the known genetic aberrations detectable by cytogenetic techniques [3], [4], [5], [6]. These AML cases are classified into a subgroup named normal karyotype AML as they lack specific markers for further classification. The variation of treatment response and prognosis of normal karyotype AML suggests that this subgroup of AML can be heterogeneous with different genetic aberrations. Indeed, mutations in several genes including *CEBPA*, *FLT3*, *IDH1*, *MLL*, and *NPM1*, and alternated expression of AF1q have been identified in the normal karyotype AML, and the potential value of these changes for clinical applications are under investigation [7], [8], [9]. Using whole genome sequencing approach, two normal karyotype AML genomes have been sequenced [10], [11]. The studies identified heterozygous, non-synonymous somatic mutations in 10 genes in the first case, and in 12 genes in the second case. Except for the known mutations in *FLT3, IDH1, and NPM1,* however, the rest novel mutations are individual case-specific but not shared within a cohort of 187 additional AML cases, of which 76 are normal karyotype AML [12]. Therefore, identification of recurrent genetic mutations in nomal karyotype AML remains a challenging task.

Genetic aberrations could be examined at the levels of genomic DNA, transcription or translation. As the genomic DNA structure in normal karyotype AML is intact as revealed by cytogenetic and genome sequencing data, an alternative approach to search for potential aberrations in normal karyotype AML could be at the level of transcription. To investigate this possibility, we used the next generation sequencing-based paired-end RNAseq method [13] to detect potential fusion transcripts in a group of normal karyotype AML cases. Similar approaches have been applied recently in multiple types of cancer with the idenification of many new fusion transcripts [14], [15], [16], [17], [18], [19]. Our comprehensive sequence data collection, analysis and experimental validation result in the identification of many novel fusion transcripts formed exclusively between the genes adjacent in the same chromosome in normal karyotype AML. Here we report our analyses and observations.

## Results

### RNAseq sequence collection and analysis

**Figure 1:**
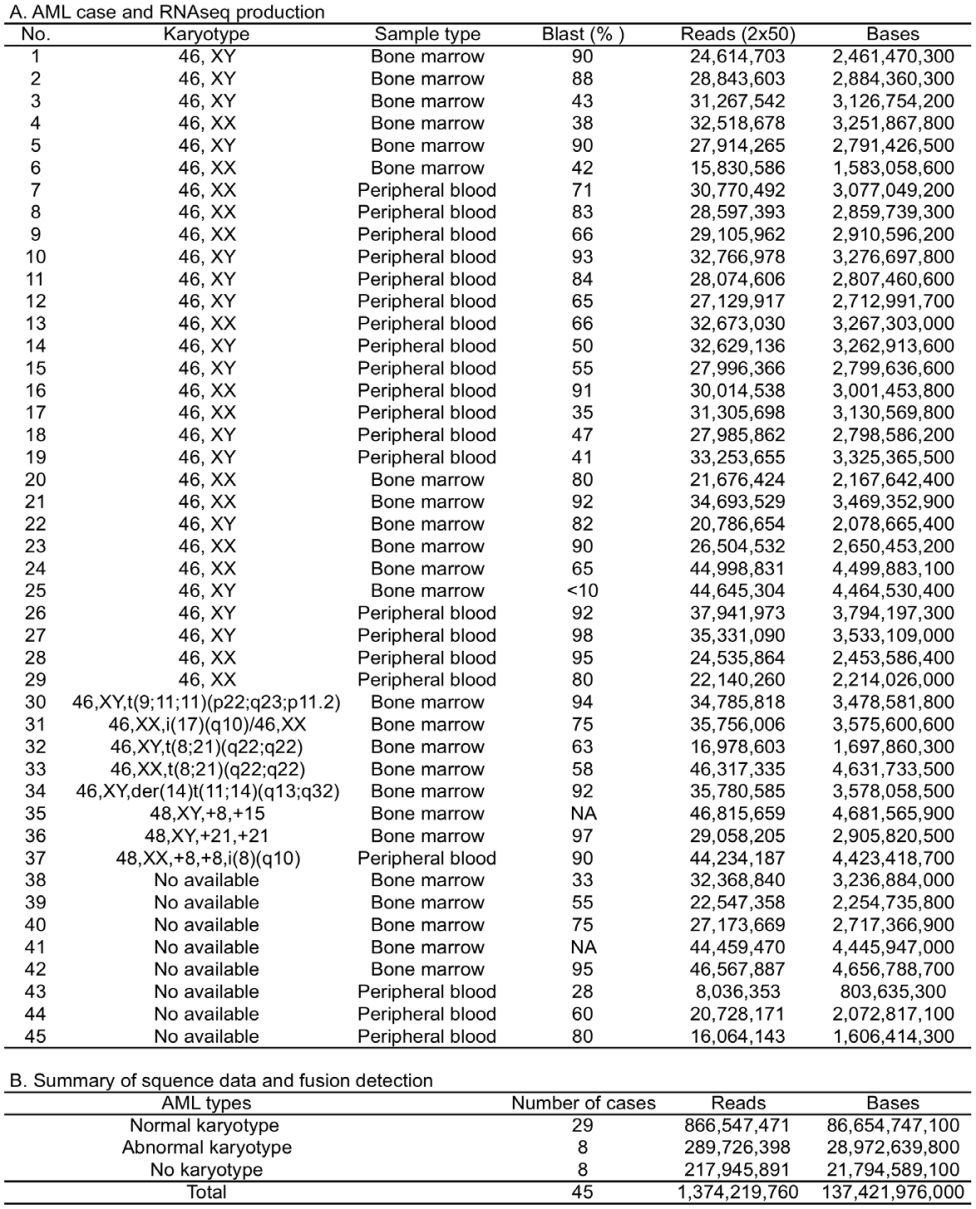
AML sample list and RNAseq data collection. A. A total of 45 AML samples were used for the analysis, including 29 normal karyotype AML, 8 abnormal karyotype AML and 8 AML without karyotype information. B. RNAseq data collected from the 45 AML cases.

Twenty-nine normal karyotype primary AML cases were used for the study. Each case was diagnosed using the standard criteria and cytogenetic analysis as normal karyotype AML in University of Michigan Medical Center. In addition, eight abnormal karyotype AML cases and eight AML cases without karyotype data were also included for the study. PolyA+ mRNA was extracted from each sample, and subjected to paired-end RNAseq sequencing (2×50). A total of 1,374,219,760 paired-end reads (137,421,976,000 bases), or on average 30,538,217 reads (3,053,821,688 bases) per sample, were collected in the study (Figure 1).

### Identification of fusion transcripts

**Figure 2:**
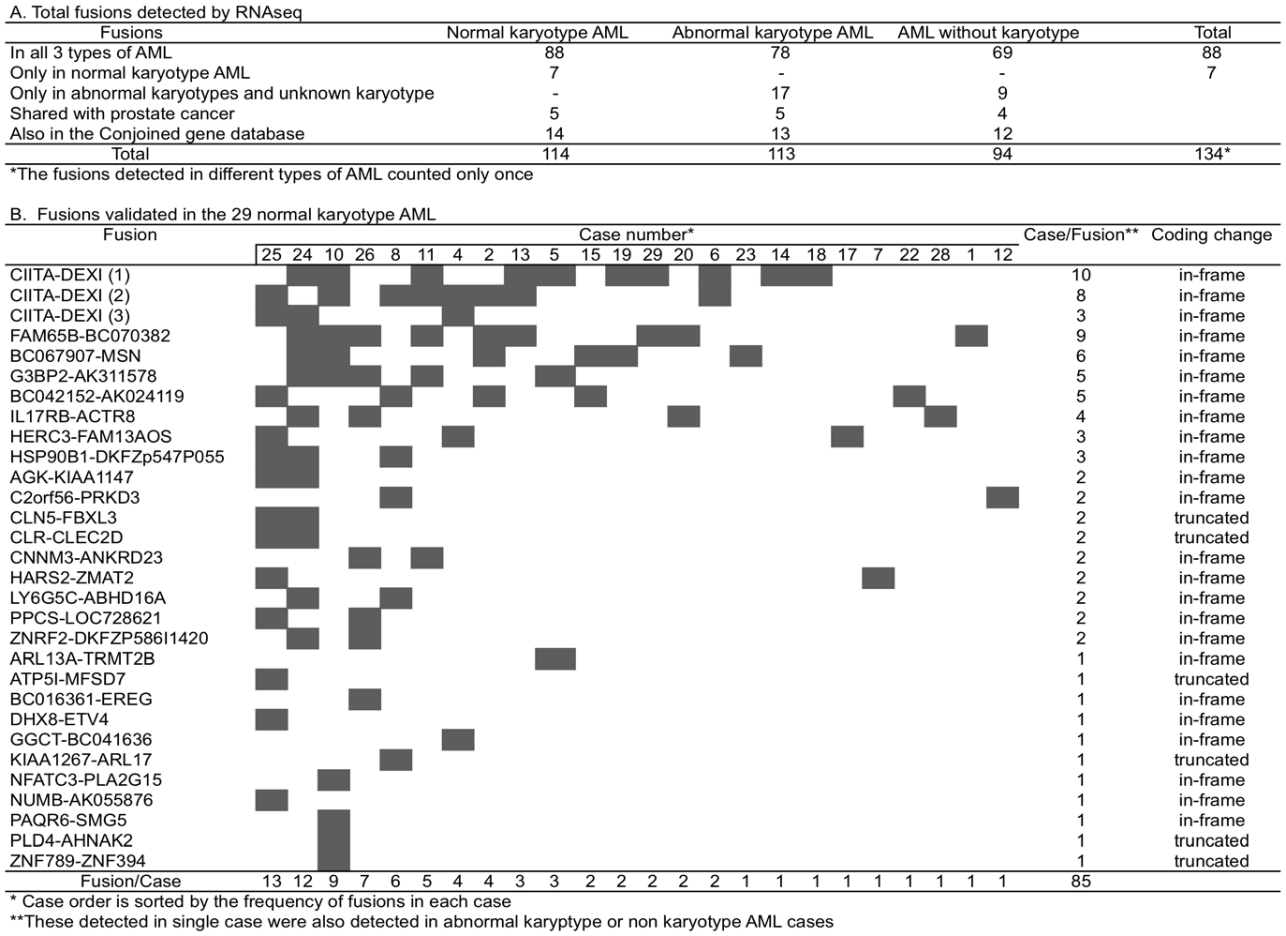
Fusion transcript information. A. Fusion transcripts identified in different types of AML. B. Validated fusion transcripts identified in normal karyotype AML.

Using the FusionSeq and deFuse programs [20], [21], we analyzed the paired-end RNAseq data to identify fusion transcripts. Three well-known fusions in AML were identified in four abnormal karyotype AML cases, including *RUNX1*-*RUNX1T1* fusion in two cases [46,XXt(8;21)(q22;q22); 46,XYt(8;21)(q22;q22)], *MLL*-*MLLT1* fusion in one case [48,XX,+8,+8,i(8)(q10)/49,idem,+i(8)(q10)] and *MLL*-*MLLT3* fusion in one case [46,XY,t(9;11;11)(p22;q23;p11.2)]. The detection of these three known fusions shows the sensitivity of the paired-end RNAseq and the mapping programs used for the analysis. Under the threshold that a fusion must be detected by at least two pairs of fusion sequences and in at least two AML cases, a total of 134 fusions were identified in the 45 AML samples used in the study (Figure 2A). In the 29 normal karyotype AML, 114 fusions were identified, of which 88 are novel fusions detected in all AML, 7 fusions [FAM65A-CTCF (6 cases), KIAA1267-ARL17 (5 cases), *GGCT-BC041636* (3 cases), *R3HDM2-INHBC* (3 cases), LY6G5C-ABHD16A (2 cases), *CRNKL1-NAA20* (2 cases), and *HSPA14-SUV39H2* (2 cases)] were detected only in normal karyotype AML. Twenty-six fusions were only detected in the abnormal karyotype AML and/or the AML without karyotype information (Figure 2A). Each fusion was distributed at different frequencies among the AML cases, with the highest one of 57 fusions detected in a normal karyotype AML case (#25, Table S1).

### Validation of fusions

**Figure 3:**
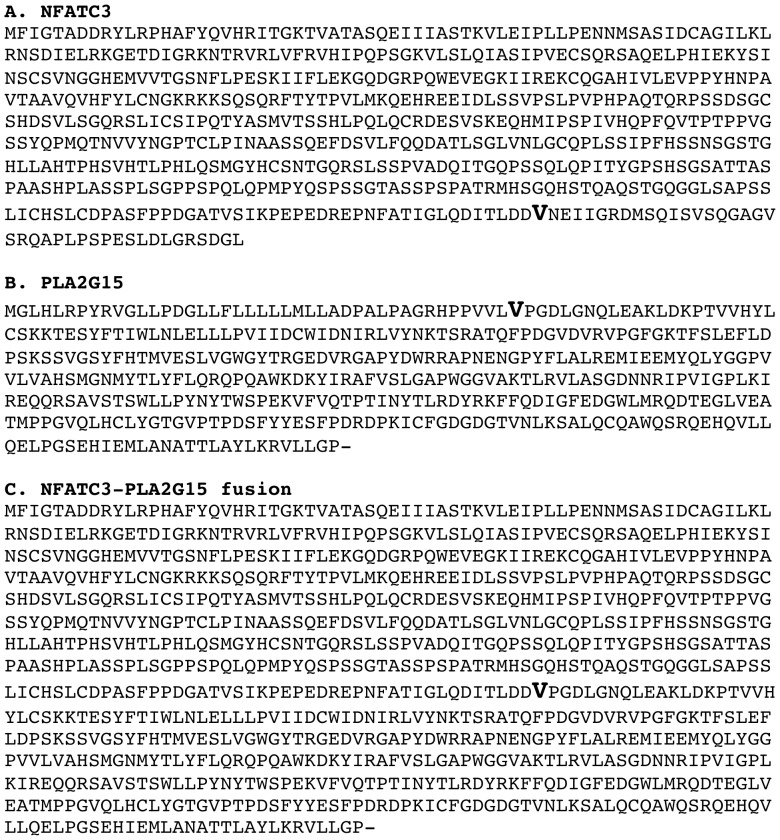
NFATC3-PLA2G15 fusion. The fusion is formed between upstream gene *NFATC3* and downstream gene *PLA2G15* in 3′-5′ tail to head orientation. In this fusion, amino acid V (GTC) is shared at the fusion point (G from *NFATC3* and TC from *PLA2G15*). A. Wild-type NFATC3 protein sequence; B. Wild-type PLA2G15 protein sequence; C. NFATC3-PLA2G15 fusion protein sequences. The bold V residue marks the fusion junction.

Based on the availability of patient RNA samples, the suitable size and base composition of the fusion sequences for primer design, and the frequency of fusion distribution in the AML cases, 66 fusions were selected for experimental validation by using antisense primers for reverse transcription, PCR amplification and Sanger sequencing. A total of 37 (56%, the relative lower rate is related with the detection of sense transcripts only by using antisense primer for reverse transcription. See below for explanation) were confirmed as true fusion transcripts in the 45 leukemia samples, of which 30 fusions were in the 29 normal karyotype AML cases (Figure 2B, Figure S1, Table S2). Of the 30 fusions, 24 maintain in-frame coding. For example, *NFATC3-PLA2G15* fusion is formed between the upstream gene *NFATC3* and the downstream gene *PLA2G15* in 3′-5′ tail to head orientation. This fusion is in-frame and the V resudue at the fusion junction is shared between the fusion partners (Figure 3). Six fusions truncated their coding structure [*CLN5-FBXL3, CLR-CLEC2D, ATP5I-MFSD7, KIAA1267-ARL17, PLD4-AHNAK2*, and *ZNF789*-*ZNF394*, Figure 2B].

### Genomic origin of fusion transcripts

**Figure 4:**
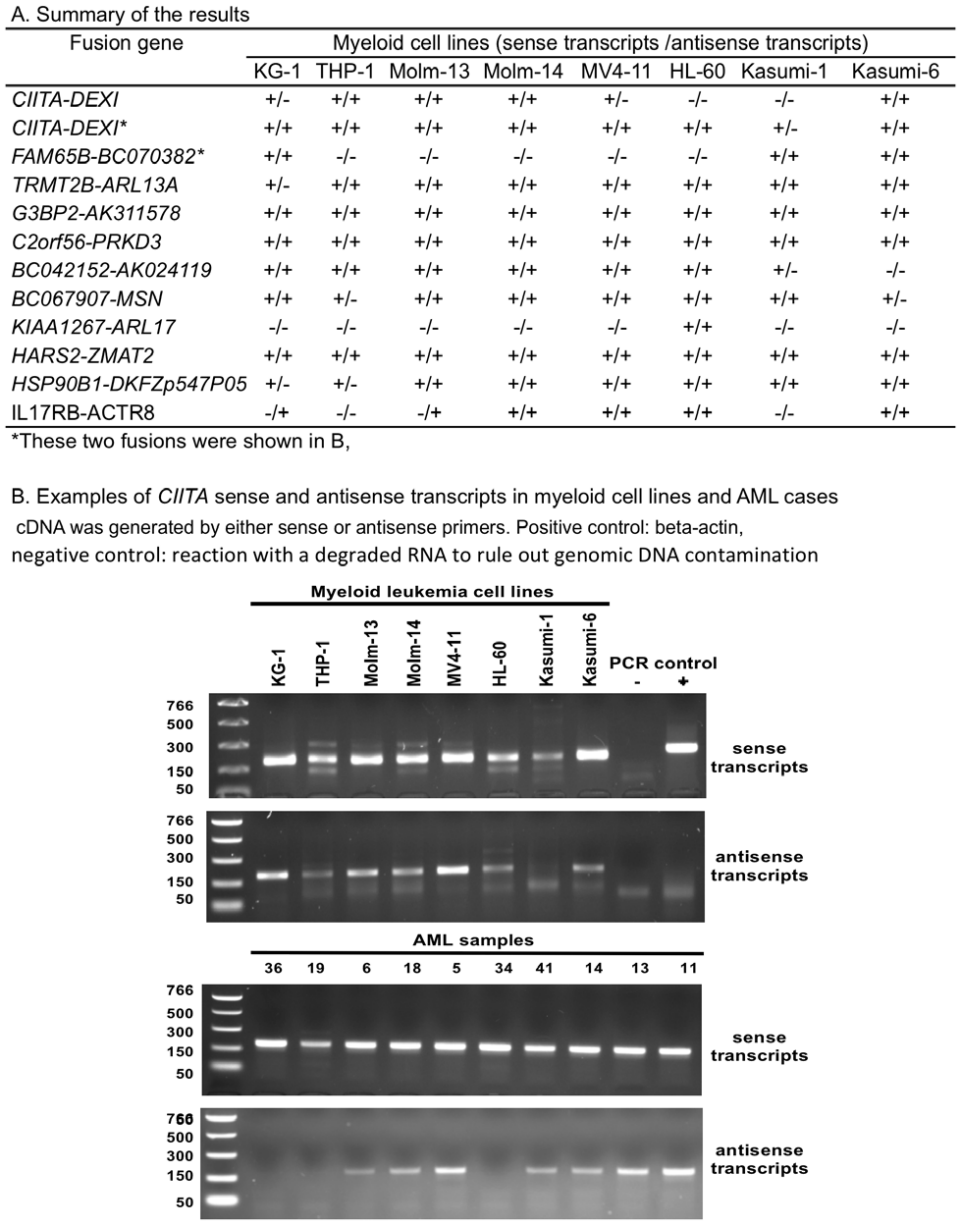
Validation of sense and antisense fusion transcripts by strand-specific RT-PCR. A. Summary for RNA samples from 8 myeloid cell lines. B. *CIITA-DEXI* sense and antisense fusion transcripts detected in myeloid cell lines and AML samples. +: positive control with beta-actin; -: netative control without RNA templates.

Mapping the fusions to the human genomic reference sequences (hg18) shows that all the detected novel fusions are formed between the genes adjacent each other in the same chromosome. Complicated orientations of upstream and downstream partner genes are present, including 3′-5′ tail to head, 5′-3′ head to tail, 3′-3′ tail to tail, and 5′-5′ head to head (Figure S2). Considering that there are two partner genes involved in each fusion, a question remains whether only one or both partner genes drove the fusion transcription. In the former case, only one strand should be used as the transcription template resulting in either sense or antisense transcripts; in the latter case, both strands should be used as transcription templates resulting in both sense and antisense transcripts. RNAseq sequences collected by the standard RNAseq method can identify the fusion junction but can not discriminate the original strand used for fusion transcription, and regular RT-PCR using only antisense primer for reverse transcription as used in our validation experiment above can only validate the transcripts from one DNA strand. To address this issue, we performed strand-specific RT-PCR using sense primer and antisense primer separately for reverse transcription. We used RNA samples from 8 myeloid leukemia-derived cell lines (KG-1, THP-1, Molm-13, Molm-14, MV4-11, HL-60, Kasumi-1, and Kasumi-6) to overcome the shortage of patient RNA samples for the analysis, and we selected 12 fusions for the test. The results confirmed that each fusion was expressed in at least one of the cell lines, and determined that 86% of fusion transcripts (68 out of 79 positive reactions in the total of 96 reactions) were transcribed from both sense and antisense strands (Figure 4A). For example, sense and antisense transcripts for one of the two *CIITA-DEXI* fusions were expressed in 7 out of 8 cell lines (Figure 4B). Testing the *CIITA-DEXI* fusion in 10 AML RNA samples show similar pattern of sense and antisense transcription that the sense fusion transcripts were expressed in all and the antisense fusion transcripts were expressed in 7 of the 10 AML samples (Figure 4B).

### Possible causes of fusion formation

**Figure 5:**
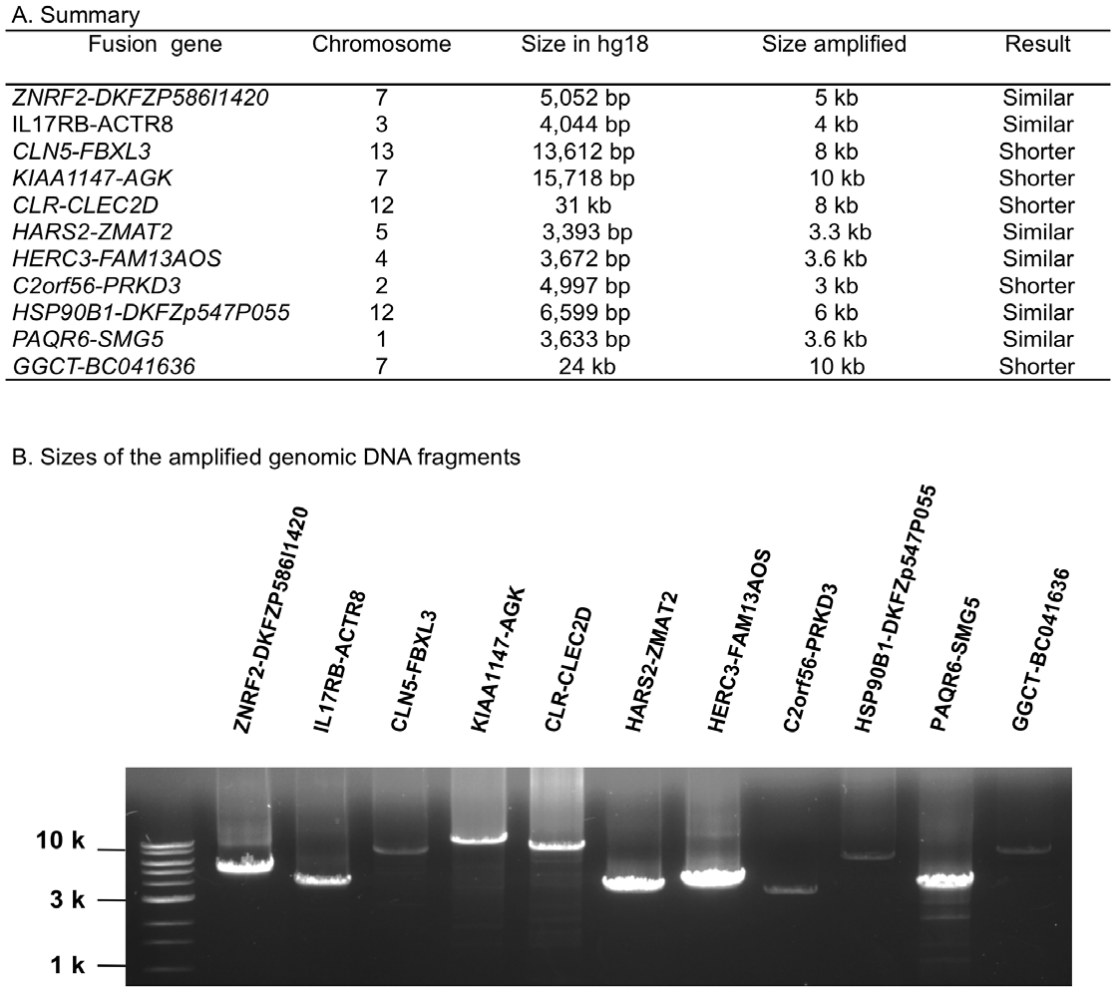
Long-range PCR results. A. Summary of the results from 11 fusion candidates. B. Size distribution of the amplified genomic DNA fragments.

Both genomic structural changes between the partner genes and post-transcriptional process can cause the formation of fusion transcripts. We used long-range PCR to amplify the mapped genomic region between the partner genes to know if there is a genomic structural change as reflected by the size difference of the amplified region comparing to the reference genome sequences. Of the 11 successfully amplified genomic DNA fragments, 6 have the sizes similar to and 5 have the sizes shorter than their corresponding regions in the reference genome sequences (Figure 5). For example, the size between *IL17RB-ACTR8* fusion is 4,044 bp in the reference genome sequences whereas the size of the amplified genomic fragment in AML is about 4 kb, suggesting that this fusion was generated by post-transcriptional process rather than genomic structural change; in the case of *CLR-CLEC2D* fusion, the size in the reference genome sequences is 31 kb but the size of the amplified genomic fragment has only 8 kb. This fusion transcript is likely caused by genomic DNA deletion between the two partner genes. The results indicate that both genomic structural change and post-transcriptional processing can contribute to the fomation of the detected fusion transcripts.

### Specificity of fusions for AML

We used several filters to determine the specificity of fusion transcripts detected in AML. A recent comprehensive study identified 800 “Conjoined genes”, which are formed between the genes adjacent in the same chromosome in the human genome [22]. As the data set is largely derived from normal human genome data, we used these as a filter to eliminate the fusions also present in normal human cells. The comparison identified and eliminated 16 such fusions from the AML fusion list (*ADSL-SGSM3, ARHGAP27-LOC201175, C11orf79-C11orf66, CS-CNPY2, ETFB-CLDND2, FLOT2-DHRS13, GALT-IL11RA, GPN3-ARPC3, MGC72080-ASNS, RAB24-MXD3, RRM2-C2orf48, SCO2-TYMP, STX16-NPEPL1, TNFAIP8L2-SCNM1, VAMP8-VAMP5, and VPS72-TMOD*4, Table S1). Fusion transcripts between adjacent genes have also been identified in multiple types of solid cancer including prostate cancer [13], ovarian cancer [15], breast cancer [16], lung cancer and colorectal cancer [18]. We compared the fusions identified in normal karyotype AML with the fusions identified by these studies. Five fusions (*XPA-NCBP1*, *HARS2-ZMAT2*, *HSP90B1-DKFZp547P055*, *IL17RB-ACTR8*, *ANKRD23*-*ANKRD39*) are identified to share with the fusions identified in prostate cancer but no fusions are shared with breast cancer, colorectal cancer, ovarian cancer, and lung cancer. Further classification of the remaining 113 fusions shows that 7 fusions (*FAM65A-CTCF, KIAA1267-ARL17, BC041636-GGCT, R3HDM2-INHBC, ABHD16A-LY6G5C, CRNKL1-NAA20, HSPA14-SUV39H2*) are present only in normal karyotype AML, 18 are present only in abnormal karyotype AML or AML without karyotype information, and 88 fusioins are shared between normal, abnormal karyotype, and no karyotype AML groups (Table S1). The fusions present only in each group have lower frequencies [2–6 (7–21%) for the 7 fusions only in the 29 normal karyotype AML, 1 to 3 (6–19%) for the 18 fusions only in the 16 abnormal karyotype AML and AML without karyotype information], but the frequencies for the fusions shared in the three AML groups are from low to high [2–30 (4–67%) for the 88 fusions in all 45 AML samples]. The frequencies of the fusion distribution in these AML groups imply that the majority of the fusions identified in AML are not related with the karyotype status of the AML cases. Although the common fusions may not be much value as markers for abnormal karyotype AML as they already have well-defined genetic markers, these fusions can be potentially very useful markers for the normal karyotype AML.

### *CIITA-DEXI* fusions

**Figure 6:**
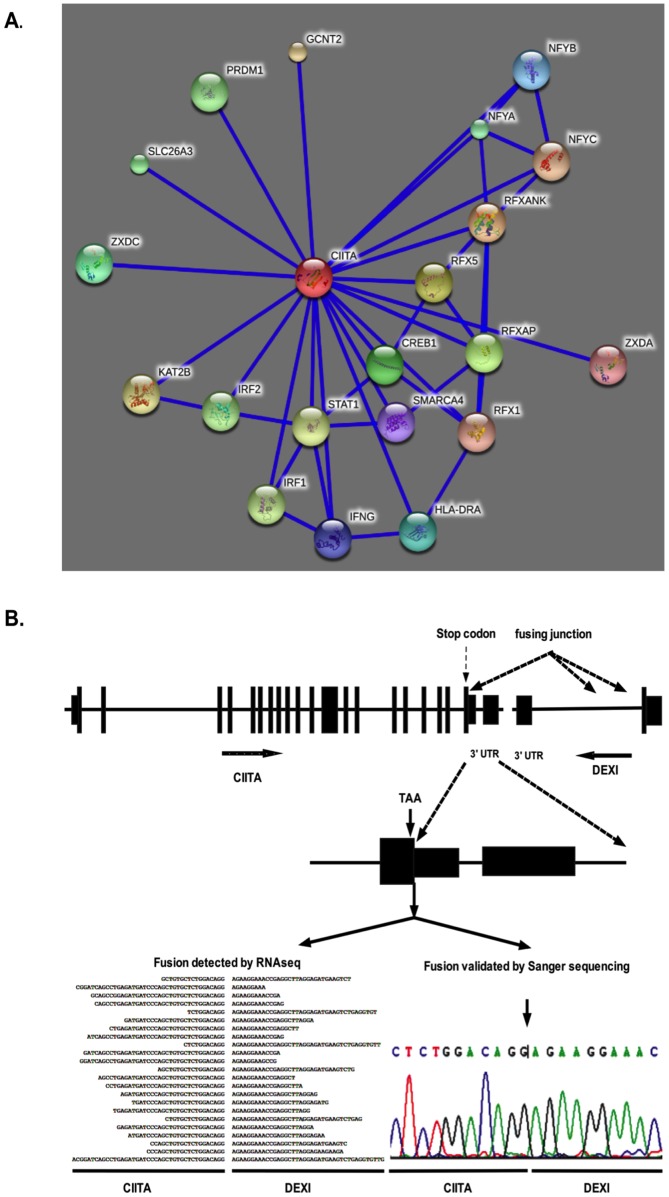
*CIITA-DEXI* fusion. A. CIITA-involved protein-protein interaction network. B. Mapping and validation of *CIITA-DEXI* fusion. Three *CIITA-DEXI* fusions were detected. In the fusion, *CIITA* preserved the coding exone till the stop codon but losed its 3′ untranslated region. This fusion was detected by 23 paired-end RNAseq sequences and validated by Sanger sequencing.

Three *CIITA-DEXI* fusion isoforms were detected in 14 of the 29 normal karyotype AML cases [Figure 2B, Figure S2]. They were formed between *CIITA* and *DEXI* in 3′–3′ tail to tail orientation in chromosome 16 [Figure 6B]. The three fusions contain the same *CIITA* part after the stop codon and fused to different locations of the same intron of *DEXI*. CIITA is a master regulator of *MHCII* gene expression, which is important for antigen-presenting cells to maintain their proper immune function. CIITA functions through interaction with multiple proteins including RFX complex (RFXANK, RFX5, and RFXAP) bound at *MHCII* promoters and other transcriptional factors (Figure 6A). Mutation of *CIITA* causes Bare Lymphocyte Syndrome, a disease unable to fight against infection [23]. DEXI is a calcium binding protein with two exons of which only one is for coding. The *CIITA-DEXI* fusion maintains the entire coding exons of *CIITA* including its stop codon but removes the entire 3′ UTR of *CIITA* (Figure 6B). This is very different from the *CIITA*-fusions identified in lymphoma, in which the *CIITA* parts are truncated in the coding region [24]. Functional means of *CIITA-DEXI* fusion in myeloleukemogenesis remains to be elucidated.

## Discussion

Fusion genes are frequently present in hematopoietic cancer, often caused by structural changes of translocation, inversion, deletion and insertion involving different chromosomes or distal regions in the same chromosome. While normal karyotype AML lacks the well-known genetic aberrations, our study shows that fusion transcripts, predominantly derived between the genes adjacent in the same chromosome, are widely present in this subgroup of AML. It is also interesting to note that the majority of the fusion transcripts detected in the normal karyotype AML are also present in the abnormal karyotype AML cases. This fact indicates that the expression of the fusion transcripts are independent events regardless the karyotype status of the AML cells.

Unlike the fusions between the adjacent genes in normal human genome, in which the partner genes are unilaterally orientated along the same chromosome and expressed most likely by the “read-through” mechanism [22], the partner genes of the fusions identified in AML consist of very heterogeneous patterns of partner gene orientation. The presence of antisense transcripts for the majority of the genomic fusion loci further complicates the determination of the original strands used for the fusion transcription.

To identify the fusion transcripts present in AML, it is important to exclude the fusion transcripts present in the normal cells of the same individual and the normal cells mixed with the AML samples (the blast rate of the AML samples used in this study is between 95% to <10%, see Figure 1). Obtaining normal cells from the same leukemic individual, e.g., skin tissue, may not be a perfect choice as evidence shows that the normal skin tissue from leukemic patients can be highly contaminated with leukemic cells [10]. To overcome this technical difficulty, we used the “conjoined genes” as the filter to exclude the AML fusions also present in normal human cells. Although this filter is not perfect solution, it is helpful to narrow down the fusion candidates associated with AML.

There are seven fusions detected only in the normal karyotype AML (*FAM65A-CTCF, KIAA1267-ARL17, BC041636-GGCT, R3HDM2-INHBC, ABHD16A-LY6G5C, CRNKL1-NAA20, HSPA14-SUV39H2*). The involved gene *CTCF, INHBC and SUV39H2* are particularly interesting. *CTCF* encodes a zinc-finger transcriptional regulator protein with 11 zinc fingers. It regulates transcription both positively and negatively. Mutations in *CTCF* have been observed in breast, prostate, and Wilms' cancers [25]. *INHBC* encodes the beta C chain of inhibin, a member of the TGF-beta superfamily involving hormone secretion [26]. The fusions of *FAM65A-CTCF* and *R3HDM2-INHBC* both removed the first exon and translational starting site of CTCF and *INHBC*, likely caused loss of the function of *CTCF and INHBC*; *SUV39H2* encodes a histone-lysine N-methyltransferase [27]. The *HSPA14-SUV39H2* fusion removed the last 3 exons of *HSPA14* and connected the remaining *HSPA14* part to the first exon of *SUV39H2*.

Five fusions (*XPA-NCBP1*, *HARS2-ZMAT2, HSP90B1-DKFZp547P055, IL17RB-ACTR8*, *ANKRD23*-*ANKRD39)* are shared between AML and prostate cancer. This raises an interesting question whether certain fusions could be common genetic aberrations contributing to different types of cancer. The *HARS2-ZMAT2* fusion was formed in-frame between the 3′ UTR of *HARS2* and the 3^rd^ exon of *ZMAT2* in 3′-5′ tail to head, sense orientation. *HARS2* (histidyl-tRNA synthetase 2) is a nuclear-encoded mitochondrial protein involving in the synthesis of histidyl-transfer RNA [28]. Mutations in *HARS2* cause Perrault syndrome with ovarian dysgenesis and hearing loss [29]. *ZMAT2* is a matrin-type 2 zinc finger protein with unknown function. The *HSP90B1-DKFZp547P055* fusion was formed between the 17^th^ of 18 exons of *HSP90B1* before its stop codon and the 2^nd^ exon of a functionally unknown gene *DKFZp547P055*, fused in a 3′-3′ tail to tail orientation between the two genes. HSP90B1 is a member of HSP90 proteins located in the endoplasmic reticulum. It is involved in folding Toll-like receptors involving in regulating innate and adaptive immunity, maintaining hematopoietic stem cell development, and potentially to be used as a vaccine for cancer treatment [30], [31], [32], [33]. The *HSP90B1-DKFZp547P055* fusion disrupted the normal structure of *HSP90B1*, and possibly led to loss of function of HSP90B1. *IL17RB-ACTR8* fusion is formed in a 3′-3′ tail to tail opposite orientation before the stop codon of *IL17RB* and the 3′ UTR of *ACTR8*. IL17RB is involved in the activation of the NF-kappaB pathway [34]. This in-frame fusion maintains the normal codon of *IL17RB*, suggesting that the fusion could be translated into the two original proteins. Prostate cells are derived from endoderm and hematopoietic cells are derived from mesoderm. Therefore, the shared fusions between prostate cancer and AML are unlikely attributed to the same developmental origin. It will be interesting to further study the biological basis of the shared fusion transcripts between the two very different cancer types. *MHCII* is generally expressed in T cells, B cells and dendritic cells involving antigen presentation [35]. While myeloid cells are not directly involved in antigen presentation, it has been observed that AML cells express many B-cell specific genes including *CIITA* [36]. It will be interesting to know if the altered *CIITA* in normal karyotype AML could contribute to its escape of the host's immune-surveillance.

Sense and antisense transcripts are commonly present for the majority of human genes [37]. Our data show that sense and antisense transcripts are also present for most of the fusions detected. The orientations of the fusion partners at genomic DNA level are very different. It will be expected that only the 3′ to 3′, tail to tail fused partners can drive the sense and antisene transcription. However, of the 12 fusions used for sense antisense detection, 6 are not in the 3′ to 3′ orientation but sense antisense transcripts were detected in each case. How were the sense and antisense transcripts expressed from these fusion loci remain to be understood.

While the biological/pathological function of many fusion transcripts identified in this study remains to be elucidated, a recent study shows that fusion transcripts formed by post-transcriptional process can have functional relevance [38]. Importantly, the high recurrence of the fusion transcripts formed between adjacent genes in AML provides a new targeting area to study disease mechanism and a rich resource to search for potential markers for clinical applications, particularly for the cancer types with intact genomic structure, as examplified by the normal karyotype AML.

## Methods

### AML samples

The study used 29 primary samples of normal karyotype AML, eight AML primary samples with abnormal karyotype AML and eight AML primary samples without karyotype information [Figure 1], of which 38 were from University of Michigan Comprehensive Cancer Center and 7 were from Allcells Inc (AllCells, CA). The use of samples from University of Michigan was approved by the University of Michigan Institutional Review Board (IRBMED #2004-1022), and written informed consent was obtained from all patients prior to enrollment.

### RNAseq process

Total RNA was extracted from each AML sample using TRIZOL reagent (Invitrogen). RNAseq library was prepared using PolyA+ mRNA isolated from each RNA sample following Illumina RNASeq protocol. Paired-end sequencing at 2×50 was performed by using Illumina GAIIx sequencer. Raw sequence data were processed by the standard GAIIx quality control program. FusionSeq [20] and deFuse [21] programs were used for fusion detection following the instructions. In FusionSeq mapping, reads from each end of a pair were mapped to the human reference genome sequences (hg18) independently. A set of filters, including PCR filter, homology filter, annotation filter, repeat filter, abnormal insert size filter and distance filter with default parameters in the program were used to remove low quality fusion candidates. Conditions were further set that a candidate fusion must be supported by at least two paired-end reads crossing the same boundary and the mapped sequences in both partner genes must contain at least 10 bp. In deFuse mapping, paired-end reads were mapped to both spliced and unspliced transcripts from Ensembl (version 62). Those mapped to the same transcript, mitochondrion and ribosome RNA were removed. The remaining paired-reads were trimmed and mapped again to the spliced and unspliced transcript sequences to remove those mapped to the same transcript. The remaining reads were clustered into contigs. Paired-end reads with only one end mapped were used to find breakpoint. The resulting fusions were further filtered based on the mapping of exon junction sequence (e.g., GT or AG) to generate the final fusion candidates. The Conjoined genes identified from the human genome were obtained from the ConjoinG database [22] (http://metasystems.riken.jp/conjoing/). The fusions identified from prostate cancer, breast cancer, ovarian cancer, lung cancer and colorectal cancer were obtained from the references [14], [15], [16], [17], [18], [19].

### AML cell lines

Eight cell lines of AML origin were used for validation study. Cell lines of KG-1, THP-1, MV4-11, HL-60, Kasumi-1 and Kasumi-6 were obtained from the American Type Culture Collection; Molm-13 and Molm-14 were provided by John Kersey (University of Minnesota). All cell lines were cultured in RPMI-1640, 10% fetal calf serum, 2 mM L-glutamine, 50 units/ml penicillin and 50 µg/ml streptomycin (Invitrogen). Total RNA was isolated from each cell line (5×10^6^ cells) using the RNeasy Protect Cell Mini Kit (QIAGEN).

### Experimental validation

Strand-specific RT-PCR and Sanger sequencing were used to validate the fusion transcripts identified by sequence mapping analysis. For each fusion selected for the test, at least one AML case was used for the validation. For primer design, sequences longer than the paired-end sequences covering the fusion were generated by extending the fusion–mapped location upstream and downstream within the exon region. Sense and antisense primers were designed using Primer3 program (http://frodo.wi.mit.edu/). Strand-specific reverse transcription was performed with either antisense primer or sense primer (2 pmole), total RNA (100 ng), M-MLV Reverse Transcriptase (2 units/reaction, Invitrogen) in a 20 µl volume at 65°C for 5 minutes, 37°C for 50 minutes, and 70°C for 15 minutes. Five µl of the resulting cDNA were used for PCR amplification in 50 µl containing sense primer (10 pmole), antisense primer (10 pmole), Taq polymerase (1.25 U, Promega), MgCl_2_ (1.5 mM) in an ABI 9700 thermal cycler under cycling conditions of 95°C 7 minutes, 38 cycles of 95°C 30 seconds, 56°C 30 seconds, and 72°C 30 seconds, and final extension at 72°C 7 minutes. PCR products were loaded on 2% agarose gels. The bands with expected size were excised from gel, purified with MinElute Gel Extraction Kit (QIAGEN), and sequenced by BigDye reagents (Life Technologies). Sequences were mapped to hg18 by Genetyx program (version 7.03, Genetyx).

### Long-range PCR

The same sense/antisense primer pairs used for RT-PCR were used to amplify the genomic regions by using a LongRange PCR kit (QIAGEN) with cycling conditions of 93°C 3 minutes, 35 cycles of 93°C 15 seconds, 55°C 30 seconds, and 68°C 4 minutes, and final extension at 68°C 7 minutes. The amplified products were checked on 0.8% agarose gels and the sizes were estimated by comparing with 1 kb DNA ladder (BioLabs).

### Protein-protein interaction analysis

String 9.0 program was used to identify the interaction proteins for CIITA [39, http://string-db.org/newstring_cgi/show_input_page.pl?UserId = RUj0Zcmw26Xa&sessionId = cVwSUCEMLeFG]. Confidence score was set at the highest level (0.900), and the interactors shown were set at no more than 20.

## Supporting Information

Figure S1 **RNAseq mapped fusion.**
(PDF)

Figure S2 **Genomic patterns of fusion partner genes.**
(PDF)

Table S1 Full fusion transcript list.
(XLS)

Table S2 Validated fusion transcript list.
(XLS)
