# Supplementary material for: New Fusion Transcripts Identified in Normal Karyotype Acute Myeloid Leukemia
Source: PLoS One. 2012 Dec 12;7(12):e51203. doi: 10.1371/journal.pone.0051203 (PMC3520980; doi:10.1371/journal.pone.0051203)
Supplement: Figure S1 — RNAseq mapped fusion. (PDF) [file pone.0051203.s001.pdf]

Supplementary figure 1. RNAseq identified fusions.

CIITA-DEXI (bp1)

Tile 1: chr16:10924643-10924683
Tile 2: chr16:10942544-10942584

CTGAGATGATCCCAGCTGTGCTCTGGACAGG	GACAAGCGAGAATCCCCC
CGGATCAGCCTGAGATGATCCCAGCTGTGCTCTGGACAGG	GACAAGCGA
GCCTGAGATGATCCCAGCTGTGCTCTGGACATG	GACAAGCGAGAATCCC
ACTGAGATGATCCCAGCTGTGCTCTGGACAGG	GACAAGCGAGAATCCCC
AGATGATCCCAGCTGTGCTCTGGACAGG	GACAAGCGAGAATCCCCCAAA
TCCCAGCTGTGCTCTGGACAGG	GACAAGCGAGAATCCCCCAAAAGCCAA
GGATCAGCCTGAGATGATCCCAGCTGTGCTCTGGACAGG	GACAAGCGAG
TGATCCCAGCTGTGCTCTGGACAGG	GACAAGCGAGAATCCCCCAAAAGC
GATGATCCCAGCTGTGCTCTGGACAGG	GACAAGCGAGAATCCCCCAAAA
GCTGTGCTCTGGACAGG	GACAAGCGAGAATCCCCCAAAAGCCAAGGTCA
CGGCCTGAGATGACCCCAGCTGTGCTCTGGACAGG	GACAAGCGAGAATC
TGTGCNCTGGACAGG	GACAAGCGAGAATCCCCCAAAAGCCAAGGTCATC
ATCCCAGCTGTGCTCTGGACAGG	GACAAGCGAGAATCCCCCAAAAGCCA
TGAGATGATCCCAGCTGTGCTCTGGACAGG	GACAAGCGAGAATCCCCCA
CTGTGCTCTGGACAGG	GACAAGCGAGAATCCCCCAAAAGCCAAGGTCAT
GCTCTGGACAGG	GACAAGCGAGAATCCCCCAAAAGCCAAGGTCATCATC
GATCAGCCTGAGATGATCCCAGCTGTGCTCTGGACAGG	GACAAGCGAGAAT
ACGGATCAGCCTGAGATGATCCCAGCTGTGCTCTGGACAGG	GACAAGCGAG
TGCTCTGGACAGG	GACAAGCGAGAATCCCCCAAAAGCCAAGGTCATCATCA
GATCCCAGCTGTGCTCTGGACAGG	GACAAGCGAGAATCCCCCAAAAGCCAA
CCCAGCTGTGCTCTGGACAGG	GACAAGCGAGAATCCCCCAAAAGCCAAGGT
GAGATGATCCCAGCTGTGCTCTGGACAGG	GACAAGCGAGAATCCCCCAAAA
TCAGCCTGAGATGATCCCAGCTGTGCTCTGGACAGG	GACAAGCGAGAATCA
CTCTGGACAGG	GACAAGCGAGAATCCCCCAAAAGCCAAGGTCATCATCAAG
AGCTGTGCTCTGGACAGG	GACAAGCGAGAATCCCCCAAAAGCCAAGGTCAT
AGCCTGAGATGATCCCAGCTGTGCTCTGGACAGG	GACAAGCGAGAATCCCA
ATGATCCCAGCTGTGCTCTGGACAGG	GACAAGCGAGAATCCCCCAAAAGCC
ACGGATCAGCCTGAGATGATCCCAGCTGTGCTCTGGACAGG	GACAAGCGAGAATCCCCCAAAAGCCAAGGTCATCATCAAGC

CIITA-DEXI (bp2)

Tile 1: chr16:10924643-10924683
Tile 2: chr16:10936473-10936513

GCTGTGCTCTGGACAGG	AGAAGGAAACCGAGGCTTAGGAGATGAAGTCT
CGGATCAGCCTGAGATGATCCCAGCTGTGCTCTGGACAGG	AGAAGGAAA
GCAGCCGGAGATGATCCCAGCTGTGCTCTGGACAGG	AGAAGGAAACCGA
CAGCCTGAGATGATCCCAGCTGTGCTCTGGACAGG	AGAAGGAAACCGAG
TCTGGACAGG	AGAAGGAAACCGAGGCTTAGGAGATGAAGTCTGAGGTGT
GATGATCCCAGCTGTGCTCTGGACAGG	AGAAGGAAACCGAGGCTTAGGA
CTGAGATGATCCCAGCTGTGCTCTGGACAGG	AGAAGGAAACCGAGGCTT
ATCAGCCTGAGATGATCCCAGCTGTGCTCTGGACAGG	AGAAGGAAACCGAG
CTCTGGACAGG	AGAAGGAAACCGAGGCTTAGGAGATGAAGTCTGAGGTGTT
GATCAGCCTGAGATGATCCCAGCTGTGCTCTGGACAGG	AGAAGGAAACCGA
GGATCAGCCTGAGATGATCCCAGCTGTGCTCTGGACAGG	AGAAGGAAGCCG
AGCTGTGCTCTGGACAGG	AGAAGGAAACCGAGGCTTAGGAGATGAAGTCTG
AGCCTGAGATGATCCCAGCTGTGCTCTGGACAGG	AGAAGGAAACCGAGGCT
CCTGAGATGATCCCAGCTGTGCTCTGGACAGG	AGAAGGAAACCGAGGCTTA
AGATGATCCCAGCTGTGCTCTGGACAGG	AGAAGGAAACCGAGGCTTAGGAG
TGATCCCAGCTGTGCTCTGGACAGG	AGAAGGAAACCGAGGCTTAGGAGATG
TGAGATGATCCCAGCTGTGCTCTGGACAGG	AGAAGGAAACCGAGGCTTAGG
CTGTGCTCTGGACAGG	AGAAGGAAACCGAGGCTTAGGAGATGAAGTCTGAG
GAGATGATCCCAGCTGTGCTCTGGACAGG	AGAAGGAAACCGAGGCTTAGGA
ATGATCCCAGCTGTGCTCTGGACAGG	AGAAGGAAACCGAGGCTTAGGAGAA
CCAGCTGTGCTCTGGACAGG	AGAAGGAAACCGAGGCTTAGGAGATGAAGTC
CCCAGCTGTGCTCTGGACAGG	AGAAGGAAACCGAGGCTTAGGAGAAGAAGA
ACGGATCAGCCTGAGATGATCCCAGCTGTGCTCTGGACAGG	AGAAGGAAACCGAGGCTTAGGAGATGAAGTCTGAGGTGTTG

FAM65B-BC070382

Tile 1: chr6:24983983-24984024
Tile 2: chr6:25150057-25150098

CGGGAGTCTGGTCGGTAGTC	GGTTGCGCTGGCGCCTCCGGATCCTGTCCCT
CCGGGAGTCTGGTCGGTAGTC	GGTTGAGCTGGCGCTTCCGGATCCTGTCCC
CTCCGGGAGTCTGGTCGGTAGTC	GGTTGAGCTGGCGCCTCCGGATCCTGTC
TTCCGGGAGTCTGGTCGGTAGTC	GGTTGAGCTGGCGCCTCCGGATCCTGTC
ATTTCCGGGAGTCTGGTCGGTAGTC	GGTTGAGCTGGCGCCTCCGGATCCTG
ACATGATTTCCGGGAGTCTGGTCGGTAGTC	GGTTGAGCTGGCGCCTCCGGA
TACCAACATGATTTCCGGGAGTCTGGTCGGTAGTC	GGTTGAGCTGGCGCCT
GTCTGGTCGGTAGTC	GGTTGAGCTGGCGCCTCCGGATCCTGTCCCTCCATG
CATGATTTCCGGGAGTCTGGTCGGTAGTC	GGTTGAGCTGGCGCCTCCGGAT
CAACATGATTTCCGGGAGTCTGGTCGGTAGTC	GGTTGAGCTGGCGCCTCCG
CGATCCTACCAACATGATTTCCGGGAGTCTGGTCGGTAGTC	GGTTGAGCTG
GTCGGTAGTC	GGTTGAGCTGGCGCCTCCGGATCCTGTCCCTCCATGGCCTG
GAGTCTGGTCGGTAGTC	GGTTGAGCTGGCGCCTCCGGATCCTGCCCCTCCA
ATGATTTCCGGGAGTCTGGTCGGTAGTC	GGTTGAGCTGGCGCCTCCGGATC
CAACATGATTTCCGGGAGTCTGGTCGGTAGTC	GGTTGAGCTGGCGCCTCCG
CCAACATGATTTCCGGGAGTCTGGTCGGTAGTC	GGTTGAGCTGGCGCCTCC
TACCAACATGATTTCCGGGAGTCTGGTCGGTAGTC	GGTTGAGCTGGCGCCT
ATCCTACCAACATGATTTCCGGGAGTCTGGTCGGTAGTC	GGTTGAGCTGGC
GGATCCTACCAACATGATTTCCGGGAGTCTGGTCGGTAGTC	GGTTGAGCTGGCGCCTCCGGATCCTGTCCCTCCATGGCCTG
TTTCCGGGAGTCTGGTCGGTAGTC	GGTTGAGCTGGCGCCTCCGGATCCTGT
GATTTCCGGGAGTCTGGTCGGTAGTC	GGTTGAGCTGGCGCCTCCGGATCCT
ACATGATTTCCGGGAGTCTGGTCGGTAGTC	GGTTGAGCTGGCGCCTCCGGA
AACATGATTTCCGGGAGTCTGGTCGGTAGTC	GGTTGAGCTGGCGCCTCCGG
CCTACCAACATGATTTCCGGGAGTCTGGTCGGTAGTC	GGTTGAGCTGGCGC
TCCGGGAGTCTGGTCGGTAGTC	GGTTGAGCTGGCGCCTCCGGATCCTGTCC
ATTTCCGGGAGTCTGGTCGGTAGTC	GGTTGAGCTGGCGCCTCCGGATCCTG
CATGATTTCCGGGAGTCTGGTCGGTAGTC	GGTTGAGCTGGCGCCTCCGGAT
CTCCGGGAGTCTGGTCGGTAGTC	GGTTGAGCTGGCGCCTCCGGATCCTGTC
CCAACATGATTTCCGGGAGTCTGGTCGGTAGTC	GGTTGAGCTGGCGCCTCC
TTCCGGGAGTCTGGTCGGTAGTC	GGTTGAGCTGGCGCCTCCGGATCCTGTC
CGGGAGTCTGGTCGGTAGTC	GGTTGAGCTGGCGCCTCCGGATCCTGTCCCT
CCGGGAGTCTGGTCGGTAGTC	GGTTGAGCTGGCGCCTCCGGATCCTGTCCC
ATGATTTCCGGGAGTCTGGTCGGTAGTC	GGTTGAGCTGGCGCCTCCGGATC
CCGGGAGTCTGGTCGGTAGTC	GGTTGAGCTGGCGCCTCCGGATCCTGTCCC
CATGATTTCCGGGAGTCTGGTCGGTAGTC	GGTTGAGCTGGCGCCTCCGGAT
TTCCTACCAACATGATTTCCGGGAGTCTGGTCGGTAGTC	GGTTGAGCTGGC
CGGGAGTCTGGTCGGTAGTC	GGTTGAGCTGGCGCCTCCGGATCCTGTCCCT
CCGGGAGTCTGGTCGGTAGTC	GGTTGAGCTGGCGCCTCCGGATCCTGTCCC
TCCGGGAGTCTGGTCGGTAGTC	GGTTGAGCTGGCGCCTCCGGATCCTGTCC
TTCCGGGAGTCTGGTCGGTAGTC	GGTTGAGCTGGCGCCTCCGGATCCTGTC
GATTTCCGGGAGTCTGGTCGGTAGTC	GGTTGAGCTGGCGCCTCCGGATCCT
ACATGATTTCCGGGAGTCTGGTCGGTAGTC	GGTTGAGCTGGCGCCTCCGGA
CCTACCAACATGATTTCCGGGAGTCTGGTCGGTAGTC	GGTTGAGCTGGCGC
GGAGTCTGGTCGGTAGTC	GGTTGAGCTGGCGCCTCCGGATCCTGTCCCTCC
CCCGGGAGTCTGGTCGGTAGTC	GGCTGAGCTGGCGCCTCCGGATCCTGTCC
TCCTACCAACATGATTTCCGGGAGTCTNGTCGGTAGTC	GGTTGAGCTGGCG
ATTTCCGGGAGTCTGGTCGGTAGTC	GGTTGAGCTGGCGCCTCCGGATCCTG
ATCCTACCAACATGATTTCCGGGAGTCTGGTCGGTAGTC	GGTTGAGCTGGC
CGGGAGTCTGGTCGGTAGTC	GGTTGAGCTGGCGCCTCCGGATCCTGTCCCT
ACATGATTTCCGGGAGTCTGGTCGGTAGTC	GGTTGAGCTGGCGCCTCCGGA
CTGCCAACATGATTTCCGGGAGTCTGGTCGGTAGTC	GGTTGAGCTGGCGCC
GGATCCTACCAACATGATTTCCGGGAGTCTGGTCGGTAGTC	GGTTGAGCTG
GGATCCTACCAACATGATTTCCGGGAGTCTGGTCGGTAGTC	GGTTGAGCTGGCGCCTCCGGATCCTGTCCCTCCATGGCCTG

ARL13A-TRMT2B

Tile 1: chrX:100115327-100115366
Tile 2: chrX:100152166-100152205

GCTGCTCTTG	CCTGAAAGTTTCTGAAACTTCAGCCTTAACAAATAGCCT
TCCGGCTTTTGTCCTCCTGCTGCTCTTG	CCTGAAAGTTTCTGAAACTTC
CGGCTTTTGTCCTCCTGCTGCTCTTG	CCTGAAAGTTTCTGAAACTTCAG
GGCTTTTGTCCTCCTGCTGCTCTTG	CCTGAAAGTTTCTGAAACTTCAGC
ATGAATCATGTTCCGGCTTTTGTCCTCCTGCTGCTCTTG	CCTGAAAGTT
CCGGCTTTTGTCCTCCTGCTGCTCTTG	CCTGAAAGTTTCTGAAACTTCAGC
CTTTTGTCCTCCTGCTGCTCTTG	CCTGAAAGTTTCTGAAACTTCAGCCTTA
TCCTGCTGCTCTTG	CCTGAAAGTTTCTGAAACTTCAGCCTTAACAAATAGC
CTCCTGCTGCTCTTG	CCTGAAAGTTTCTGAAACTTCAGCCTTAACAAATA
TTTTGTCCTCCTGCTGCTCTTG	CCTGAAAGTTTCTGAAACTTCAGCCTTAA
TTCCGGCTTTTGTCCTCCTGCTGCTCTTG	CCTGAAAGTTTCTGAAACTTCA
ATGAATCATGTTCCGGCTTTTGTCCTCCTGCTGCTCTTG	CCTGAAAGTTTCTGAAACTTCAGCCTTAACAAATAGCCT

G3BP2-AK311578

Tile 1: chr4:76806216-76806257
Tile 2: chr4:76868483-76868524

CTTCTCCATAACCATTTCTTTGCTGCACAATGTCAAATG	CTGTCACGTTGG
GGCTTCTCCATAACCATTTCTTTGCTGCACAATGTCAAATG	CTGTCACGTT
CTTTGCTGCACAATGTCAAATG	CTGTCACGTTGGCCAAAGGAAAATTTCAC
CTCCATAACCATTTCTTTGCTGCACAATGTCAAATG	CTGTCACGTTGGCCA
CATAACCATTTCTTTGCTGCACAATGTCAAATG	CTGTCACGTTGGCCAAAG
ATTTCTTTGCTGCACAATGTCAAATG	CTGGTGTTTAGATCTAGGAATTCCT
GGCTTCTCCATAACCATTTCTTTGCTGCACAATGTCAAATG	CTGGTGTTTAGATCTAGGAATTCCTTTCAGCAGGTTTGAAG

C2orf56-PRKD3

Tile 1: chr2:37329032-37329073
Tile 2: chr2:37333732-37333773

TCATATACTGCAG	CACTGCAGATGACAATCTACGAAGAACAAGTTACACAG
AGGAAACACATTTCATATACTGCAG	CACTGCAGATGACAATCTACGAAGAA
CAAGAAATCAAAATAAAGGAAACACATTTCATATACTGCAG	CACTGCAGATGACAATCTACAAAGAACAAGTTACACAGCAA
TATACTGCAG	CACTGCAGATGACAACCTACGAAGAACAAGTTACACAGCAA
ATATACTGCAG	CACTGCAGATGACAATCTACGAAGAACAAGTTACACAGCA
CACATTTCATATACTGCAG	CACTGCAGATGACAATCTACGAAGAACAAGTT
ACACATTTCATATACTGCAG	CACTGCAGATGACAATCTACGAAGAACAAGT
TAAAGGAAACACATTTCATATACTGCAG	CACTGCAGATGACAATCTACGAA
AATCAAAATAAAGGAAACACATTTCATATACTGCAG	CACTGCAGATGACAA
AGAAATCAAAATAAAGGAAACACATTTCATATACTGCAG	CACTGCAGATGA
AACACATTTCATATACTGCAG	CACTGCAGATGACAATCTACAAAGAACAAG
AAGAAATCAAAATAAAGGAAACACATTTCATATACTGCAG	CACTGCAGATG
GAAACACATTTCATATACTGCAG	CACTGCAGATGACAATCTACAAAGAACA
CAAGAAATCAAAATAAAGGAAACACATTTCATATACTGCAG	CACTGCAGATGACAATCTACAAAGAACAAGTTACACAGCAA

BC042152-AK024119

Tile 1: chr19:47328979-47329020
Tile 2: chr19:47351698-47351739

ATCAAGCAAGCCCTGCGCCTATCTGCAG	GCCGGATCTCTGGATCTCCCCAG
CGCAAGCCCTGCGCCTATCTGCAG	GCCGGATCTCTGGATCTCCCCAGCCTC
GGAAGATCAAGCAAGCCCTGCGCCTATCTGCAG	GCCGGATCTCTGGATCTC
CGCGGCAGGGAAGATCAAGCAAGCCCTGCGCCTATCTGCAG	GCCGGATCTCTGGATCTCCCCAGCCTCCAGATCTCAGTGTG

BC067907-MSN

Tile 1: chrX:64725176-64725217
Tile 2: chrX:64853404-64853445

GCAAAACAACCAA	ATCAGTGTGCGTGTGACCACCATGGATGCAGAGCTGGA
ACCATGCAAAACACCCAA	ATCAGTGTGCGTGTGACCACCATGGATGCAGAG
CTTAACTGCCAGTGTTCGTGGACCATGCAAAACAACCAA	ATCAGTGTGCGT
CCCTTAACTGCCAGTGTTCGTGGACCATGCAAAACAACCAA	ATCAGTGTGCGTGTGACCACCATGGATGCAGAGCTGGAGTT
CAAAACAACCAA	ATCAGTGTGCGTGTGACCACCATGGATGCAGAGCTGGAG
CCCTTAACTGCCAGTGTTCGTGGACCATGCAAAACAACCAA	ATCAGTGTGCGTGTGACCACCATGGATGCAGAGCTGGAGTT
GGACCATGCAAAACAACCAA	ATCAGTGTGCGTGTGACCACCATGGATGCAG
GTTCGTGGACCATGCAAAACAACCAA	ATCAGTGTGCGTGTGACCACCATGG
ACCATGCAAAACAACCAA	ATCAGTGTGCGTGTGACCACCATGGATGCAGAG
CGTGGACCATGCAAAACAACCAA	ATCAGTGTGCGTGTGACCACCATGGATG
CCAGTGTTCGTGGACCATGCAAAACAACCAA	ATCAGTGTGCGTGTGACCAC
CAAAACAACCAA	ATCAGTGTGCGTGTGACCACCATGGATGCAGAGCTGGAG
CCATGCAAAACAACCAA	ATCAGTGTGCGTGTGACCACCATGGATGCAGAGC
TTCGTGGACCATGCAAAACAACCAA	ATCAGTGTGCGTGTGACCACCATGGA
TGTTCGTGGACCATGCAAAACAACCAA	ATCAGTGTGCGTGTGACCACCATG
CCCTTAACTGCCAGTGTTCGTGGACCATGCAAAACAACCAA	ATCAGTGTGCGTGTGACCACCATGGATGCAGAGCTGGAGTT

KIAA1267-ARL17

Tile 1: chr17:41786010-41786051
Tile 2: chr17:41527741-41527782

TCTCCACACAGAAA	CCTTATTAGCACGTATCTGTTTGTAAATGTCTGTTTG
TATATTCTACTGTCTCCACACAGAAA	CCTTATTAGCACGTATCTGTTTGTA
TTTATATTCTACTGTCTCCACACAGAAA	CCTTATTAGCACGTATCTGTTTG
CGAAGGTGTTATTTTTATATTCTACTGTCTCCACACAGAAA	CCTTATTAGC
ACTGTCTCCACACAGAAA	CCTTATTAGCACGTATCTGTTTGTAAATGTCTG
GAAGGTGTTATTTTTATATTCTACTGTCTCCACACAGAAA	CCTTATTAGCA
CGAAGGTGTTATTTTTATATTCTACTGTCTCCACACAGAAA	CCTTATTAGCACGTATCTGTTTGTAAATGTCTGTTTGCTGA

HARS2-ZMAT2

Tile 1: chr5:140058558-140058599
Tile 2: chr5:140060586-140060627

ATTTGAAGTGCCACGGAACGTTGTCAAGA	GACAAAAAACTTGGACTTTCGC
ACGGAACGTTGTCAAGA	GACAAAAAACTTGGACTTTCGCCGAAAGTGGGAC
CCACGGAACGTTGTCAAGA	GACAAAAAACTTGGACTTTCGCCGAAAGTGGG
GCCACGGAACGTTGTCAAGA	GACAAAAAACTTGGACTTTCGCCGAAAGTGG
GGCAAATTTGAAGTGCCACGGAACGTTGTCAAGA	GACAAAAAACTTGGACT
AGTGCCACGGAACGTTGTCAAGA	GACAAAAAACTTGGACTTTCGCCGAAAG
CTGCAGAGGCAAATTTGAAGTGCCACGGAACGTTGTCAAGA	GACAAAAAACTTGGACTTTCGCCGAAAGTGGGACAAAGATG

HSP90B1-DKFZp547P055

Tile 1: chr12:102865296-102865337
Tile 2: chr12:102871292-102871333

CAGATGAAGGAGAAGAAACAGCAAA	GATGAGCTGAGCTGGACAACTAAAAG
AACAGATGAAGAAGAAGAAACAGCAAA	GATGAGCTGAGCTGGACAACTAAA
GGGAACAGATGAAGAAGAAGAAACAGCAAA	GATGAGCTGAGCTGGACAACT
GAAATGGATGTGGGAACAGATGAAGAAGAAGAAACAGCAAA	GATGAGCTGA
GAAATGGATGTGGGAACAGATGAAGAAGAAGAAACAGCAAA	GATGAGCTGAGCTGGACAACTAAAAGCACCTGTAGCAGTTC

IL17RB-ACTR8

Tile 1: chr3:53874044-53874085
Tile 2: chr3:53877739-53877780

```
                    CAGTGTGTGCGATGGTA  CCAAGAAGCTTGTTTTTGGTCTTCGGCAGTGACA
                 CAACAGTGTGTGCGATGGTA  CCAAGAAGCTTGTTTTTGGTCTTCGGCAGTG
               GTCAACAGTGTGTGCGATGGTA  CCAAGAAGCTTGTTTTTGGTCTTCGGCAG
                          GTGCGATGGTA  CCAAGAAGCTTGTTTTTGGTCTTCGGCAGTGACATTTCCT
                         TGTGCGATGGTA  CCAAGAAGCTTGTTTTTGGTCTTCGGCAGTGACATTTCC
  TCCTTCTTTCCAATGACGTCAACAGTGTGTGCGATGGTA  CCAAGAAGCTTG
              CGTCAACAGTGTGTGCGATGGTA  CCAAGAAGCTTGTTTTTGGTCTTCGGCA
    CTTCTTTCCAATGACGTCAACAGTGTGTGCGATGGTA  CCAAGAAGCTTGTT
CTTCCTTCTTTCCAATGACGTCAACAGTGTGTGCGATGGTA  CCAAGAAGCTTGTTTTTGGTCTTCGGCAGTGACATTTCCTC
```

ANKRD23-CNNM3

Tile 1: chr2:96862966-96863007
Tile 2: chr2:96869463-96869504

```
                         CCAGGTGGATCCTCTT  ATCCTGTGCGTTCAGGTGGGCGCCACACTCGATGA
AGAGCAGTGAGGGAGGGTTCATGAACCAGGTGGATCCTCTT  ATCCTGTGCG
AGAGCAGTGAGGGAGGGTTCATGAACCAGGTGGATCCTCTT  ATCCTGTGCGTTCAGGTGGGCGCCACACTCGATGAGGTGCT
```

PPCS-LOC728621

Tile 1: chr1:42695568-42695609
Tile 2: chr1:42711380-42711421

```
                      CATCTGGGGGCCCACTGCAGG  GAAAAGTTCAGTTAGAAGACATACTTCACC
           CAAGATCCAGTCATCTGGGGGCCCACTGCAGG  GAAAAGTTCAGTTAGAAGA
          ACAAGATCCAGTCATCTGGGGGCCCACTGCAGG  GAAAAGTTCAGTTAGAAG
GCCTGAACACAAGATCCAGTCATCTGGGGGCCCACTGCAGG  GAAAAGTTCAGTTAGAAGACATACTTCACCATCTTGAAAAA
```

HERC3-FAM13AOS

Tile 1: chr4:89848392-89848433
Tile 2: chr4:89850282-89850323

```
                               AAGAATCCAT  CCCTTTACAGAAAACAACTGACCAATCCTTATGAGACCAGA
                              AAAGAATCCAT  CCCTTTACAGAAAACAACTGACCAATCCTTATGAGACCAG
                              AAAGAATCCAT  CCCTTTACAGAAAACAACTGACCAATCCTTATGAGACCAG
               GAAAATTCTACATGATTAAAGAATCCAT  CCCTTTACAGAAAACAACTGACC
              AGAAAATTCTACATGATTAAAGAATCCAT  CCCTTTACAGAAAACAACTGAC
                              TAAAGAATCCAT  CCCTTTACAGAAAACAACTGACCAATCCTTATGAGACCG
                            ATTAAAGAATCCAT  CCCTTTACAGAAAACAACTGACCAATCCTTATGAGAC
                    ATTCTACATGATTAAAGAATCCAT  CCCTTTACAGAAAACAACTGACCAATC
                  AAATTCTACATGATTAAAGAATCCAT  CCCTTTACAGAAAACAACTGACCAA
        GGAGTAAGAAAATTCTACATGATTAAAGAATCCAT  CCCTTTACAGAAAACA
                       CTACATGATTAAAGAATCCAT  CCCTTTACAGAAAACAACTGACCAATCCTT
                        TACATGATTAAAGAATCCAT  CCCTTTACAGAAAACAACTGACCAATCCTTA
CATCATGGAGTAAGAAAATTCTACATGATTAAAGAATCCAT  CCCTTTACAGAAAACAACTGACCAATCCTTATGAGACCAGA
```

PAQR6-SMG5

Tile 1: chr1:154483129-154483170
Tile 2: chr1:154485982-154486023

```
                        GGTACCTCCACGTTGA  CCTCAACACTCCAAAAGCCAGCCCCTTCAGGTCTT
 GCTTGAGACTGAGCATGGTGGCCTGGTACCTCCACGTTGA  CCTCAACACTC
                              TCCACGTTGA  CCTCAACACTCCAAAAGCCAGCCCCTTCAGGTCTTCAGTCC
          TGAGCATGGTGGCCTGGTACCTCCACGTTGA  CCTCAACACTCCAAAAGCCA
           GAGCATGGTGGCCTGGTACCTCCACGTTGA  CCTCAACACTCCAAAAGCCAG
AGCTTGAGACTGAGCATGGTGGCCTGGTACCTCCACGTTGA  CCTCAACACTCCAAAAGCCAGCCCCTTCAGGTCTTCAGTCC
```

ZNF789-ZNF394

Tile 1: chr7:98922479-98922520
Tile 2: chr7:98934272-98934313

CTCTTACGGTCCATAAACAGTGTC ACTCGGCGGAACTGTGCTCCCGGAATC
TTACGGTCCATAAACAGTGTC ACTCGGCGGAACTGTGCTCCCGGAATCCTT
TCAGCTCTTACGGTCCATAAACAGTGTC ACTCGGCGGAACTGTGCTCCCGG
CTTCAGACAGCGGTCAGCTCTTACGGTCCATAAACAGTGTC ACTCGGCGGAACTGTGCTCCCGGAATCCTTCTGCGCACTCT

CLR-CLEC2D

Tile 1: chr12:9700978-9701019
Tile 2: chr12:9724785-9724826

AGCAGCTTCCACCATGCCCCTGGAAAAACT GTTGTCTGCATTCAAAAGAGC
ATCAGCAGCAGCTTCCACCATGCCCCTGGAAAAACT GTTGTCTGCATTCAA
GTGGAATCAGCAGCAGCTTCCACCATGCCCCTGGAAAAACT CAATAAGAGC
GTGGAATCAGCAGCAGCTTCCACCATGCCCCTGGAAAAACT CAATAAGAGCTAACTGCCATCAAGAGCCATCAGTATGTCTT

GGCT-BC041636

Tile 1: chr7:30506780-30506821
Tile 2: chr7:30530506-30530547

GGGAATTGCCAAAGTCAAGCTTAAAAT CATCCTCAGGTGAGTGGCAACCAC
TGGGAATTGCCAAAGTCAAGCTTAAAAT CATCCTCAGGTGAGTGGCAACCA
CTTGGGAATTGCCAAAGTCAAGCTTAAAAT CATCCTCAGGTGAGTGGCAAC
ACTTGTTTTGCCTTGGGAATTGCCAAAGTCAAGCTTAAAAT CATCCTCAGGTGAGTGGCAACCACCAGAAGTGGCTGTTTCA

CLN5-FBXL3

Tile 1: chr13:76474145-76474186
Tile 2: chr13:76487545-76487586

TGATGATCAACTGAACACGTTTC TGCTGGAGAGACATGAGGACAGCTGCTC
CGTCCTTTAAGTATTTGATGATCAACTGAACACGTTTC TGCTGGAGAGACA
ACTGAACACGTTTC TGCTGGAGAGACATGAGGACAGCTGCTCATTTTCAAC
TTGATGATCAACTGAACACGTTTC TGCTGGAGAGACATGAGGACAGCTGCT
GTATTTGATGATCAACTGAACACGTTTC TGCTGGAGAGACATGAGGACAGC
CAAGGTCCTTTAAGTATTTGATGATCAACTGAACACGTTTC TGCTGGAGAGACATGAGGACAGCTGCTCATTTTCAACAGCT

AGK-KIAA1147

Tile 1: chr7:140999111-140999152
Tile 2: chr7:141010440-141010481

GGGAGCTGCAGTTCTTCTGTGATC AGCACGAAGAGGTCCTCTTCACAAGGG
AGCTGCAGTTCTTCTGTGATC AGCACGAAGAGGTCCTCTTCACAAGGGTTG
GGTGAAACTGCTCCCCAGGAAGCTGCAGTTCTTCTGTGATC AGCACGAAGAGGTCCTCTTCACAAGGGTTGTAGTCTTCTTC

ZNRF2-DKFZP586I1420

Tile 1: chr7:30372398-30372437
Tile 2: chr7:30377094-30377133

GTCATCTACATAG GTATTTTCCTGTTAAATAAGATATCAAAAGCATCAATC
CTTAATAGTCATCTACATAG GTATTTTCCTGTTAAATAAGATATCAAAAGC
ATAGTCATCTACATAG GTATTTTCCTGTTAAATAAGATATCAAAAGCATCA
CCCTTAATAGTCATCTACATAG GTATTTTCCTGTTAAATAAGATATCAAAA
TGAAAATGCTGCACATCCCTTAATAGTCATCTACATAG GTATTTTCCTGTTAAATAAGATATCAAAAGCATCAATCAC

BC016361-EREG

Tile 1: chr4:75414846-75414885
Tile 2: chr4:75449731-75449770

```
                   AAAGGGAGACCTCATCT  CTGATATTTCCAGTGTCAGAGGGACACAGCCA
             TGGGCAACAAAGGGAGACCTCATCT  CTGATATTTCCAGTGTCAGAGGGA
AATTTCAGACCAGCTGGGCAACAAAGGGAGACCTCATCT  CTGATATTTCCAGTGTCAGAGGGACACAGCCAACGTGGG
```

NFATC3-PLA2G15

Tile 1: chr16:66783137-66783178
Tile 2: chr16:66840692-66840733

```
                               TTTAGATGAT  GTCCCTGGTGATTTGGGTAACCAACTGGAAGCCAAGCTGGA
                         CATCACTTTAGATGAT  GTCCCTGGTGATTTGGGTAACCAACTGGAAGCCAA
ACTTTGCAACCATTGGTCTGCAGGACATCACTTTAGATGAT  GTCCCTGGTGATTTGGGTAACCAACTGGAAGCCAAGCTGGA
```

LY6G5C-ABHD16A

Tile 1: chr6:31759055-31759096
Tile 2: chr6:31762934-31762975

```
                              GAGTGCTCCTA  CCCCATTCTTCCATAATGAGTCCCAGTTGGTCCCTAGAGG
                ACGGTGTCCCTTCTGAGTGCTCCTA  CCCCATTCTTCCATAATGAGTCCCAG
             ATGACGGTGTCCCTTCTGAGTGCTCCTA  CCCCATTCTTCCATAATGAGTCC
GCGACAGCAGCAGATGACGGTGTCCCTTCTGAGTGCTCCTA  CCCCATTCTTCCATAATGAGTCCCAGTTGGTCCCTAGAGGT
```

PLD4-AHNAK2

Tile 1: chr14:104469693-104469734
Tile 2: chr14:104474544-104474585

```
       GCAAGTTCATGGTCACGGAGAAGGCAGCCTACA  TAGAGCTGCTTAGTAGAG
               ATGGTCACGGAGAAGGCAGCCTACA  TAGAGCTGCTTAGTAGAGACCAGGAG
           GTTCATGGTCACGGAGAAGGCAGCCTACA  TAGAGCTGCTTAGTAGAGACCA
 CACCACAGCAAGTTCATGGTCACGGAGAAGGCAGCCTACA  TAGAGCTGCTT
GAACCACAGCAAGTTCATGGTCACGGAGAAGGCAGCCTACA  TAGAGCTGCTTAGTAGAGACCAGGAGTGTGTGGGAGGAAAG
```

DHX8-ETV4

Tile 1: chr17:38957039-38957080
Tile 2: chr17:38969306-38969347

```
                        CTTGTGCAGGGACATGG  GACCTCTACTCACGGTTTTCTGAATGGAAATCAG
             GTGGGTGAGCATCTTGTGCAGGGACATGG  GACCTCTACTCACGGTTTTCTG
TGGGAAGGTGGAGTGGGTGAGCATCTTGTGCAGGGACATGG  GACCTCTACTCACGGTTTTCTGAATGGAAATCAGGAACAAA
```

ATP5I-MFSD7

Tile 1: chr4:657152-657193
Tile 2: chr4:666572-666613

```
                              AGGTAATCTG  TCCAGTCAAGTGGATCCTCCCCCTGCTGGCAGGTGGACAAG
                             TAGGTAATCTG  TCCAGTCAAGTGGATCCTCCCCCTGCTGGCAGGTGGACAA
TATCCTCCTCTCCTCTTCTGCCCGAGGTTTTAGGTAATCTG  TCCAGTCAAGTGGATCCTCCCCCTGCTGGCAGGTGGACAAG
```

NUMB-AK055876

Tile 1: chr14:72999086-72999127
Tile 2: chr14:72892088-72892129

```
                              CCAAGGTACT  TAACCGGGAAGCTACATTTTCCGGTGCGAACGCCTTCTTCA
                            GGCCAAGGTACT  TAACCGGGAAGCTACATTTTCCGGTGCGAACGCCTTCTT
CGGGAAGTGCCACGCTGCTTTCCACGCTGGAACAAGGTACT  TAACCGGGAAGCTACATTTTCCGGTGCGAACGCCTTCTTCA
```
