# Supplementary material for: New Fusion Transcripts Identified in Normal Karyotype Acute Myeloid Leukemia
Source: PLoS One. 2012 Dec 12;7(12):e51203. doi: 10.1371/journal.pone.0051203 (PMC3520980; doi:10.1371/journal.pone.0051203)

Supplementary figure 2. Genomic patterns of fusion between partner genes. Each fusion transcript sequence was mapped to hg18. The genomic region covering the two fusion partner genes was cut directly from the mapped genomic region to maintain the gene structure, orientation and distance between the fusion partners. The left arrow in dot lines is the end of 5' fusion transcript sequence and the right arrow is the beginning of the 3' fusion transcript sequence.

1. CIITA-DEXI

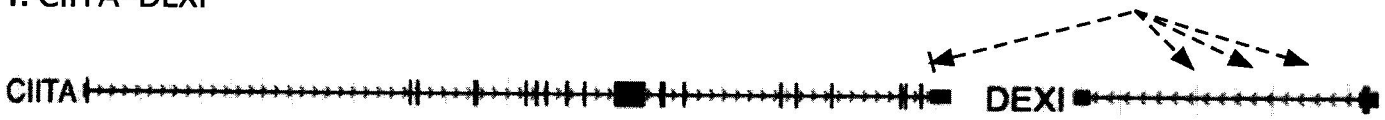

2. FAM65B-BC070382

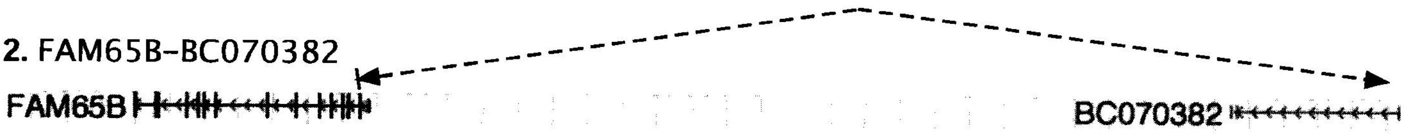

3. ARL13A-TRMT2B

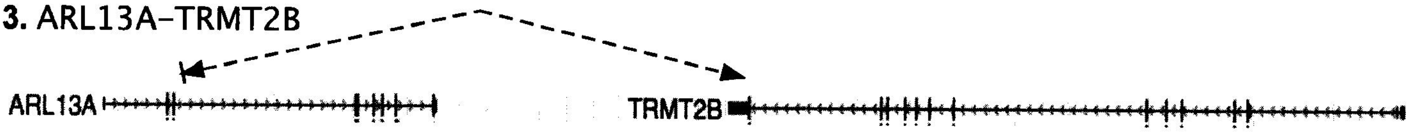

4. G3BP2-AK311578

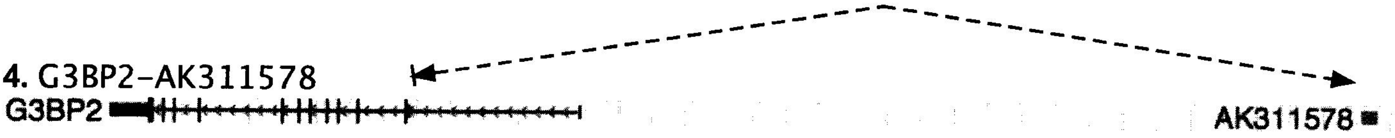

5. C2orf56-PRKD3

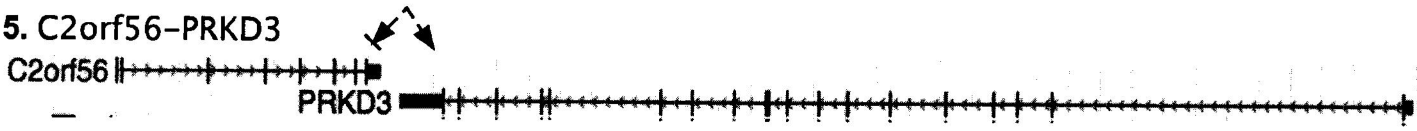

6. BC042152-AK024119

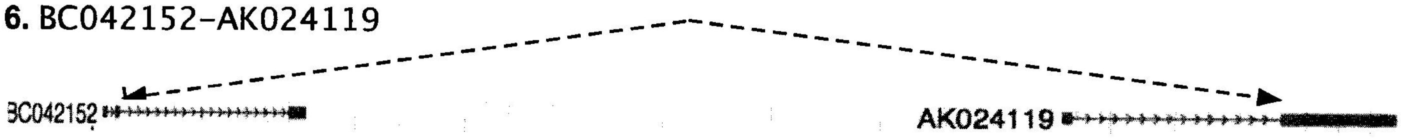

7. BC067907-MSN|MSN/ALK

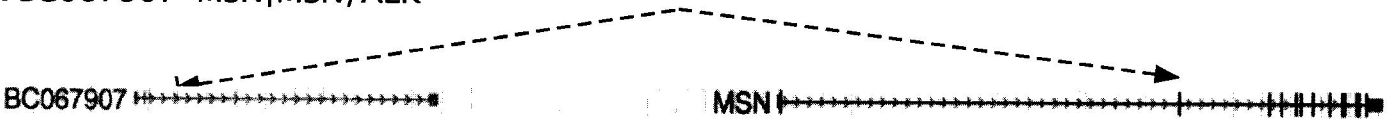

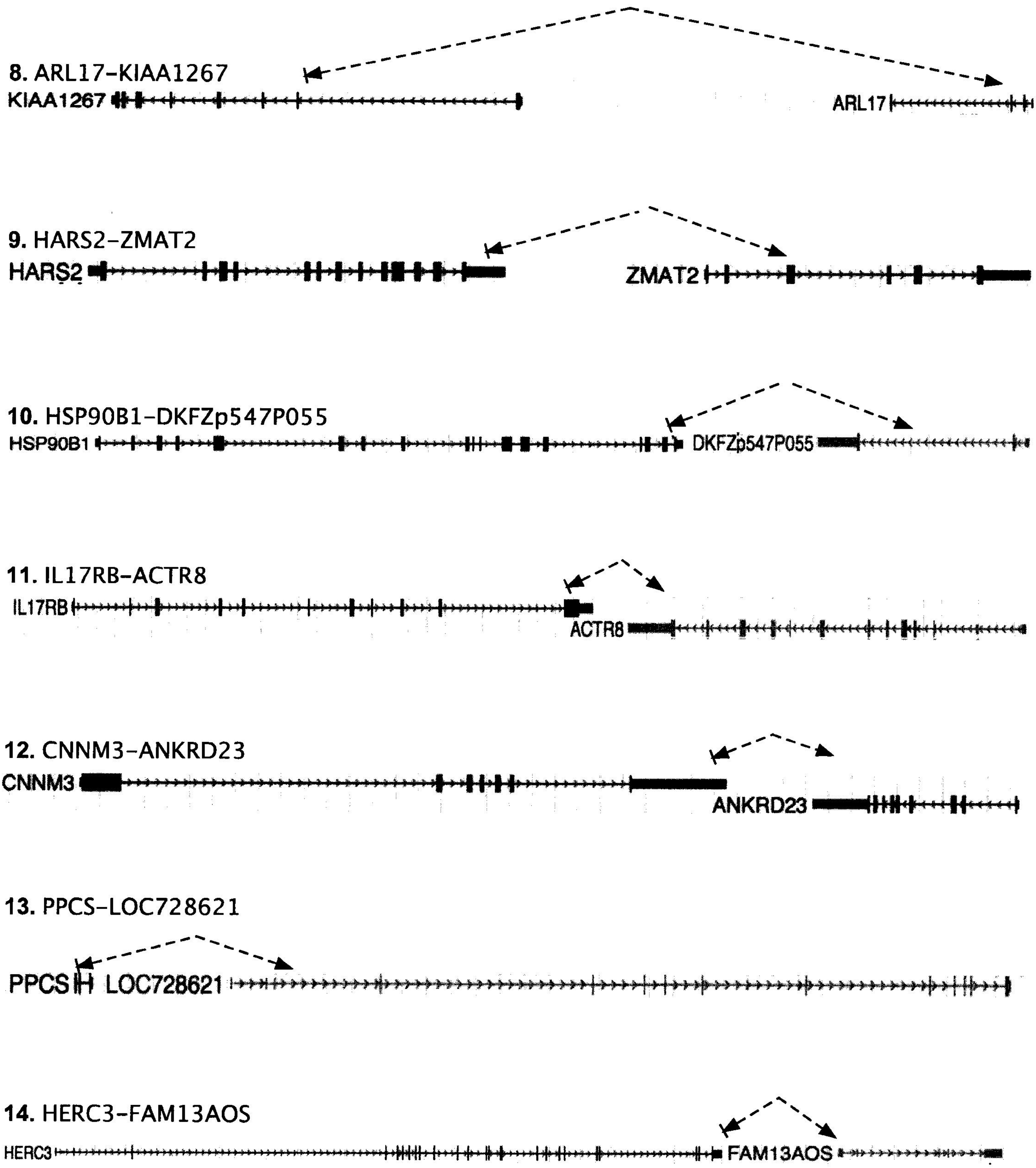

8. ARL17-KIAA1267
KIAA1267
ARL17
9. HARS2-ZMAT2
HARS2
ZMAT2
10. HSP90B1-DKFZp547P055
HSP90B1
DKFZp547P055
11. IL17RB-ACTR8
IL17RB
ACTR8
12. CNNM3-ANKRD23
CNNM3
ANKRD23
13. PPCS-LOC728621
PPCS
LOC728621
14. HERC3-FAM13AOS
HERC3
FAM13AOS

15. PAQR6-SMG5

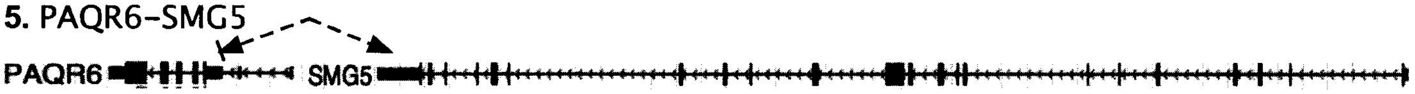

16. ZNF789-ZNF394

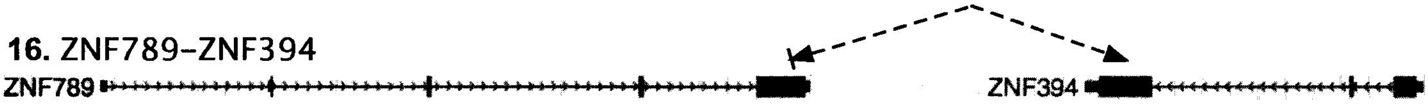

17. CLR-CLEC2D

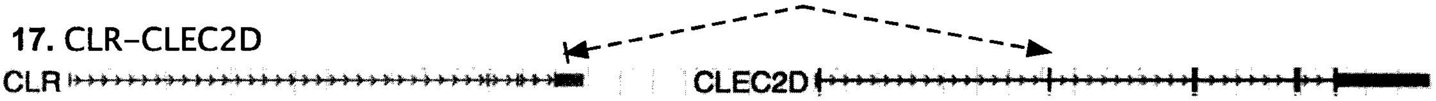

18. GGCT-BC041636

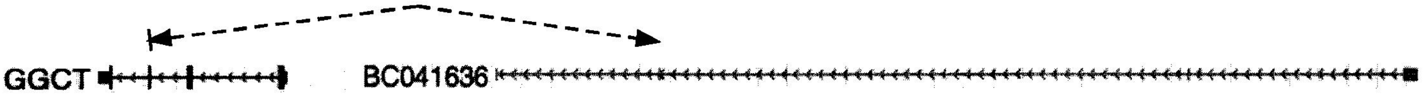

19. CLN5-FBXL3

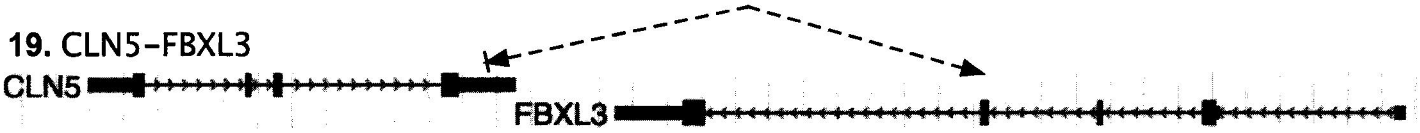

20. AGK-KIAA1147

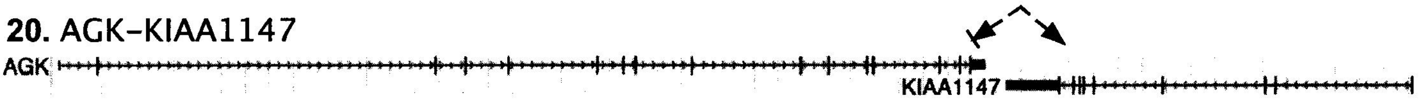

21. ZNRF2-DKFZP586I1420

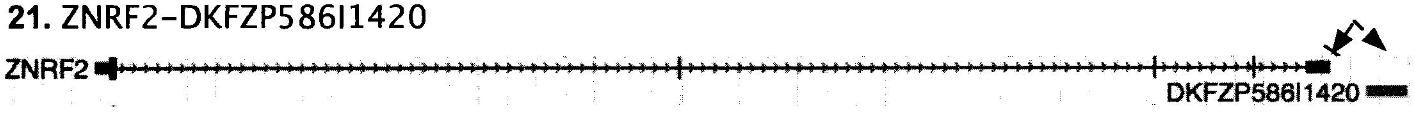

22. BC016361-EREG

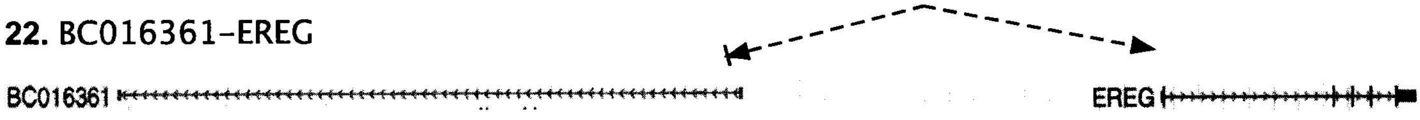

23. NFATC3-PLA2G15

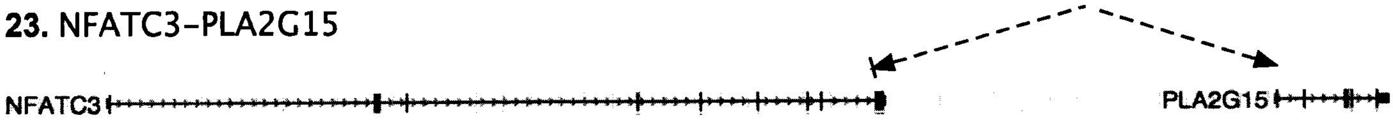

24. LY6G5C-ABHD16A

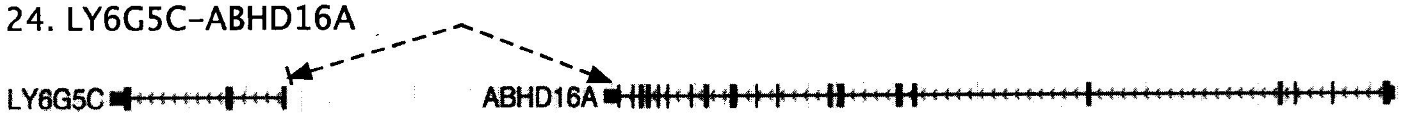

25. PLD4-AHNAK2

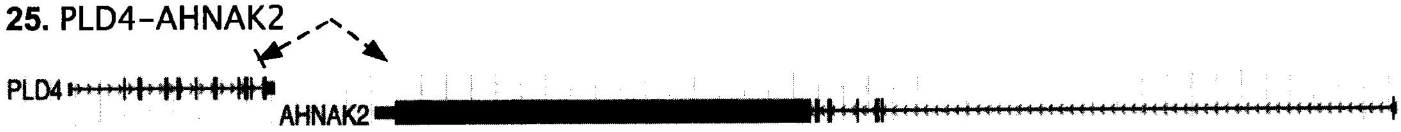

26. DHX8-ETV4

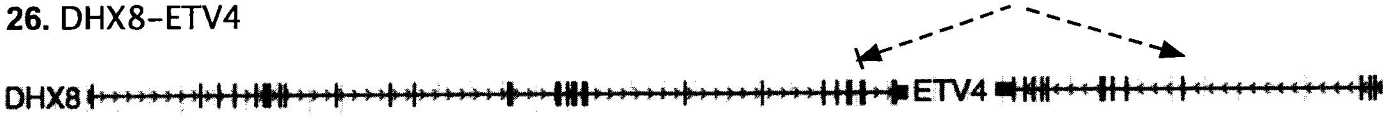

27. ATP5I-MFSD7

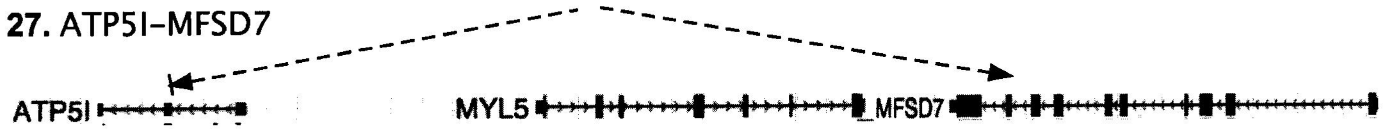

28. NUMB-AK055876

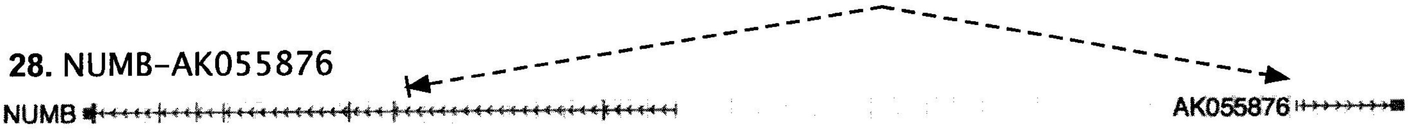

Supplement: Figure S2 — Genomic patterns of fusion partner genes. (PDF) [file pone.0051203.s002.pdf]
